# The histone H3K27 demethylase SlJMJ4 promotes dark- and ABA-induced leaf senescence in tomato

**DOI:** 10.1093/hr/uhab077

**Published:** 2022-01-19

**Authors:** Xiaochun Ding, Dandan Zhang, Dachuan Gu, Zhiwei Li, Hanzhi Liang, Hong Zhu, Yueming Jiang, Xuewu Duan

**Affiliations:** Guangdong Provincial Key Laboratory of Applied Botany, South China Botanical Garden, Chinese Academy of Sciences, Guangzhou 510650, China; Guangdong Provincial Key Laboratory of Applied Botany, South China Botanical Garden, Chinese Academy of Sciences, Guangzhou 510650, China; Center of Economic Botany, Core Botanical Gardens, Chinese Academy of Sciences, Guangzhou 510650, China; Guangdong Provincial Key Laboratory of Applied Botany, South China Botanical Garden, Chinese Academy of Sciences, Guangzhou 510650, China; Center of Economic Botany, Core Botanical Gardens, Chinese Academy of Sciences, Guangzhou 510650, China; Guangdong Provincial Key Laboratory of Applied Botany, South China Botanical Garden, Chinese Academy of Sciences, Guangzhou 510650, China; University of Chinese Academy of Sciences, Beijing 100049, China; Guangdong Provincial Key Laboratory of Applied Botany, South China Botanical Garden, Chinese Academy of Sciences, Guangzhou 510650, China; University of Chinese Academy of Sciences, Beijing 100049, China; Guangdong Provincial Key Laboratory of Applied Botany, South China Botanical Garden, Chinese Academy of Sciences, Guangzhou 510650, China; Center of Economic Botany, Core Botanical Gardens, Chinese Academy of Sciences, Guangzhou 510650, China; Guangdong Provincial Key Laboratory of Applied Botany, South China Botanical Garden, Chinese Academy of Sciences, Guangzhou 510650, China; Center of Economic Botany, Core Botanical Gardens, Chinese Academy of Sciences, Guangzhou 510650, China; Guangdong Provincial Key Laboratory of Applied Botany, South China Botanical Garden, Chinese Academy of Sciences, Guangzhou 510650, China; Center of Economic Botany, Core Botanical Gardens, Chinese Academy of Sciences, Guangzhou 510650, China

## Abstract

Leaf senescence is a highly-programmed developmental process during the plant life cycle. ABA plays an important role in leaf senescence. However, the mechanism underlying ABA-mediated leaf senescence, particularly the upstream epigenetic regulatory network, remains largely unclear. Here, we demonstrated that SlJMJ4, a Jumonji C (jmjC) domain-containing protein in tomato (*Solanum lycopersicum*), specifically demethylates di- and trimethylations of lysine 27 of histone H3 (H3K27) *in vitro* and *in vivo*. Overexpression of *SlJMJ4* results in a premature senescence phenotype and promotes dark- and ABA-induced leaf senescence in tomato. Under dark conditions, SlJMJ4-promoted leaf senescence is associated with upregulated expression of transcription factors (*SlORE1* and *SlNAP2*) and senescence-associated genes (*SlSAG113* and *SlSAG12*) via removal of H3K27me3. In response to ABA, overexpression of *SlJMJ4* increases its binding at the loci of *SlORE1*, *SlNAP2*, *SlSAG113*, *SlSAG12*, *SlABI5*, and *SlNCED3* and decreases their H3K27me3 levels, thereby activating their expression and mediating ABA-induced leaf senescence in tomato. Taken together, these results demonstrate that SlJMJ4 plays a positive role in leaf senescence in tomato and functions in ABA-induced leaf senescence by binding to many key genes related to ABA synthesis and signaling, transcription regulation, and senescence, thus promoting their H3K27me3 demethylation.

## Introduction

Senescence is the last stage in the plant life cycle. Plant senescence results in cell, tissue, organ, or even organism death [1]. Leaf senescence is characterized by chlorophyll degradation, reduced photosynthesis, and nutrient remobilization, which is critical for crop fitness and productivity. Leaf photosynthesis is essential for maximizing the carbohydrate level in seeds or fruit, and delaying senescence therefore facilitates increases in yield. In addition, efficient senescence is beneficial for maximizing stored nutrients [2]. A better understanding of the regulatory mechanism of leaf senescence has high economic relevance for decreasing yield losses.

Leaf senescence is a complicated programmed process controlled by environmental and endogenous signals that involves several layers of regulation, such as transcriptional/post-transcriptional regulation, translational/post-translational regulation, and epigenetic regulation [3]. Transcriptional regulation has crucial roles in leaf senescence. Transcription factors from the NAC [4] and WRKY [5] families have emerged as important regulators of leaf senescence in Arabidopsis and rice. Other transcription factors, such as the MYB [6], bHLH [7], bZIP [8], and AP2/EREBP [9] families, are also implicated in the regulation of leaf senescence. These transcription factors constitute complex regulatory networks with hormone signaling pathways that control the expression of senescence-associated genes (*SAGs*), thus regulating leaf senescence [10]. In recent years, great attention has been paid to the functions of epigenetic modifications, such as DNA methylation, chromatin remodeling, and histone modification, in the regulation of leaf senescence [11–14]. Yuan et al*.* [11] reported that DEMETER-like protein 3 (DML3) activates several SAGs by DNA demethylation in Arabidopsis, thereby regulating leaf longevity. Chen *et al.* [12] found that histone deacetylase HDA9 interacts with WRKY53 to promote the onset of leaf senescence. Cho et al. [13] revealed that loss of *DRD1* and *DDM1*, two SWI2/SNF2-like chromatin-remodeling proteins, postponed Arabidopsis leaf senescence. In addition, REF6 promotes the recruitment of BRM (an SWI2/SNF2-type ATPase) to target numerous SAGs [14]. Therefore, different epigenetic mechanisms are implicated in leaf senescence modulation.

Histone methylation, an important epigenetic marker, plays roles in different biological processes, such as maintenance of genome integrity and transcriptional regulation. It is written via histone methyltransferases and eliminated by histone demethylases [15]. In general, histone lysine demethylases can be classified into two types, Jumonji C (JmjC) domain-containing proteins (JMJs) and lysine-specific demethylase 1 (LSD1). LSD1 family proteins act only on mono- and dimethylated lysine, whereas JMJs show demethylase activity towards mono-, di-, and trimethylated lysine. JMJs represent the majority of histone lysine demethylases and catalyze the demethylation of lysine via oxidation, with α-ketoglutarate and Fe (II) iron as required cofactors. JMJs have been reported to regulate flowering [16], circadian rhythm [17], fruit ripening [18], stress response [19], and seed germination [20]. Recently, two important studies revealed that JMJs are involved in the regulation of Arabidopsis leaf senescence [21, 22]. REF6/JMJ12, an H3K27 demethylase, accelerates the premature activation of leaf senescence by binding to regulators of NON-YELLOWING 1 (NYE1) and directly upregulating the transcription of chlorophyll degradation genes and *SAGs* [21]. JMJ16, a specific H3K4 demethylase, negatively regulates leaf senescence in Arabidopsis by removing H3K4me3 at *WRKY53* and *SAG201* loci [22]. Based on these studies, leaf senescence is closely associated with histone modification. However, it remains unclear how histone modifications regulate leaf senescence in other plants.

Tomato, an economically important horticultural crop, is used as a model system to investigate the growth and ripening of fleshy fruit. However, there is little research on the regulation of leaf senescence in tomato. Previous studies have shown that leaf senescence in tomato is accompanied by decreased H3K27me3 levels and increased *SAG* expression [23], implying that H3K27me3 demethylases may be involved in the regulation of tomato leaf senescence. Here, we demonstrated that SlJMJ4 is an H3K27 demethylase that positively regulates dark- and ABA-induced leaf senescence in tomato. Overexpression of *SlJMJ4* reduces H3K27me3 levels in relation to the upregulated expression of *SAGs* and genes related to ABA synthesis and signaling. These results illustrate a new possible mechanism by which the H3K27 demethylase SlJMJ4 is involved in dark- and ABA-induced leaf senescence in tomato.

## Results

### Expression of *JMJ* genes in young and senescent leaves of tomato

**Figure 1:**
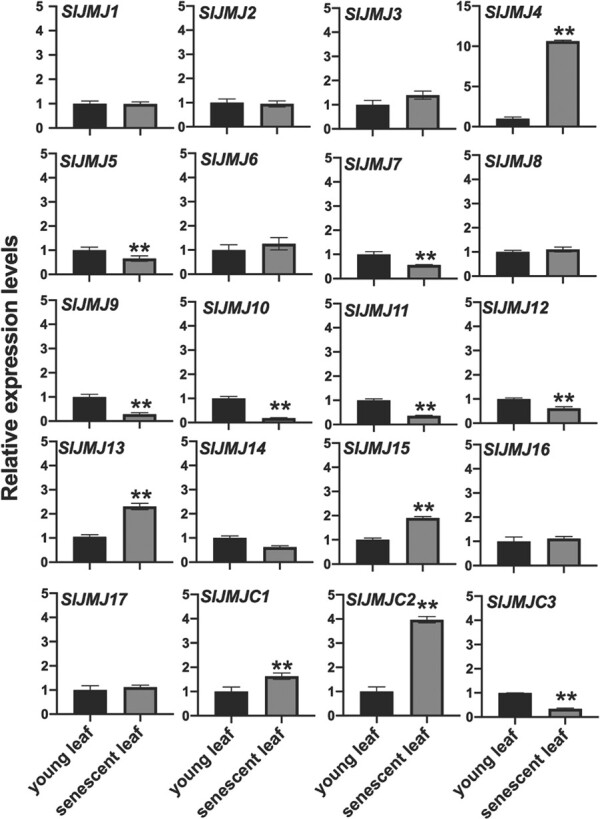
**Expression of *SlJMJ* genes in young and senescent tomato leaves from 2-month-old plants.** Young leaves were obtained from the top part of the stem, and senescent leaves were obtained from the bottom part of the stem. *ACTIN* was used as the reference gene. The values are shown as the mean ± SE of three biological replicates (Student’s *t*-test; ^**^P < 0.01).

In tomato, the JMJ family has 20 members, which can be categorized into five subfamilies, KDM4/JHDM3 (SlJMJ1, SlJMJ2, SlJMJ3, SlJMJ4), KDM5/JAR2D1 (SlJMJ5, SlJMJ6, SlJMJ7, SlJMJ8), JMJD6 (SlJMJ9, SlJMJ10, SlJMJ11), KDM3/JHDM2 (SlJMJ12, SlJMJ13, SlJMJ14, SlJMJ15, SlJMJ16, SlJMJ17), and JMJC/DOG (SlJMJC1, SlJMJC2, SlJMJC3) [18]. We compared the expression levels of all 20 *SlJMJs* in young and senescent leaves and found that several *SlJMJ* genes, including *SlJMJ4*, *SlJMJ13*, *SlJMJ15*, *SlJMC1*, and *SlJMJC2*, were significantly upregulated in senescent leaves compared with young leaves. Among these genes, SlJMJ4 showed the highest degree of upregulation (Fig. 1). These results imply that SlJMJ4 may have an important function in leaf senescence. Therefore, we chose to further elucidate the possible role of SlJMJ4 in regulating tomato leaf senescence.

### Bioinformatic analysis and subcellular localization of the SlJMJ4 protein

Sequence analyses showed that SlJMJ4 harbors a zf-C5HC2 domain, a highly conserved JmjC domain, and a JmjN domain (Fig. S1a). Based on the KDM4 subfamily sequences of its human, Arabidopsis, and rice homologs, we determined that SlJMJ4 had high homology to AtJMJ13 and OsJMJ706 (Fig. S1b). AtJMJ13 is an H3K27me3 demethylase and a photoperiod- and temperature-dependent flowering inhibitor [24], whereas OsJMJ706 has H3K9me2/3 demethylase activity and is implicated in the regulation of rice flowering [25]. Sequence alignment of homologous proteins revealed that tomato SlJMJs contain conserved Fe (II)- and α-KG-binding amino acids within the cofactor binding site (Fig. S1c), which are required for catalyzing demethylation via a hydroxylation reaction [18]. Subcellular localization analysis showed that SlJMJ4 was located in the nucleus (Fig. S1d), consistent with a possible role in the regulation of chromatin structure. Together, these results imply that SlJMJ4 may be capable of H3K27 or H3H9 demethylation and may play a positive role in gene activation.

### SlJMJ4 has specific H3K27me3/2 demethylase activity *in vivo* and *in vitro*

**Figure 2:**
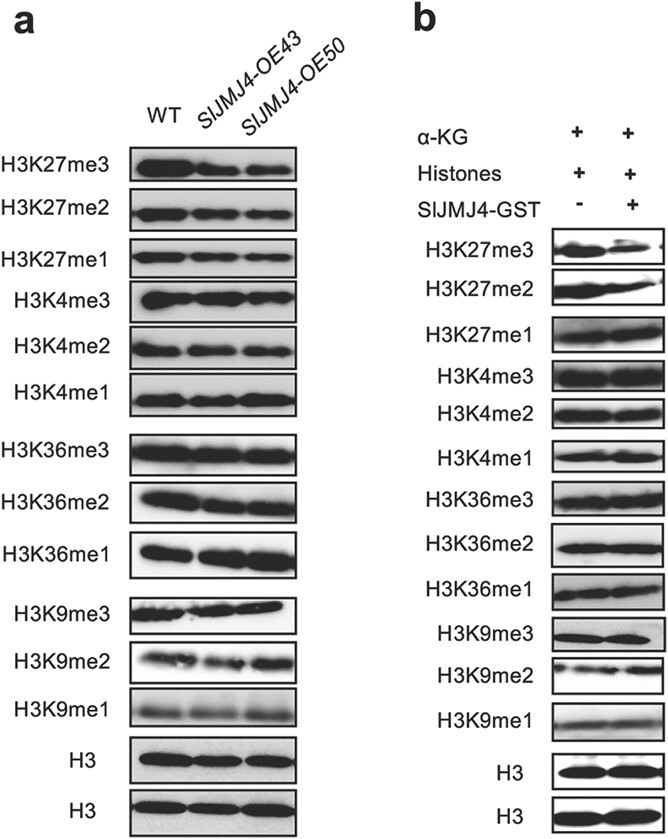
**SlJMJ4 has specific H3K27me3/2 demethylase activity *in vivo* and *in vitro*. a** Global histone methylation profiles of leaves from 2-month-old wild-type (WT) and *SlJMJ4-OE* transgenic plants. Methylation-specific antibodies were used to determine the global histone methylation status. Anti-H3 was used as a control. **b** SlJMJ4-GST exhibited H3K27me3/2 demethylase activity *in vitro*. SlJMJ4-GST was incubated with calf thymus histones, and the histone methylation status was determined with the use of specific antibodies *in vitro*. The reaction without SlJMJ4-GST was used as the control.

To confirm the site of SlJMJ4 action, we analyzed the histone demethylase activity of SlJMJ4 *in vivo* and *in vitro*. For *in vivo* analysis, we compared the histone methylation profiles of WT and *SlJMJ4-OE* plants using two independent lines, *SlJMJ4-OE43* and *SlJMJ4-OE50*. Western blotting analysis showed that the di- and trimethylation levels at H3K27 were clearly lower in *SlJMJ4-OE43* and *SlJMJ4-OE50* plants than in the WT (Fig. 2a). However, no differences were observed in the levels of H3K27me1, H3K9me1/2/3, H3K4me1/2/3, or H3K36 me1/2/3 between the WT and *SlJMJ4-OE* plants. For *in vitro* analysis, calf thymus type II-A histone was used as the substrate, and Fe(NH_4_)_2_(SO_4_)_2_, α-ketoglutarate, and ascorbate were used as the coenzymes of the enzymatic reaction. As shown in Fig. 2b, SlJMJ4-GST recombinant protein reduced H3K27me2/3 levels, yet it did not affect the levels of H3K27me1, H3K9me1/2/3, H3K4me1/2/3, or H3K36me1/2/3 *in vitro*. Taken together, these results revealed that SlJMJ4 is a specific H3K27me2/3 demethylase in tomato.

### SlJMJ4 is related to leaf senescence in tomato

**Figure 3:**
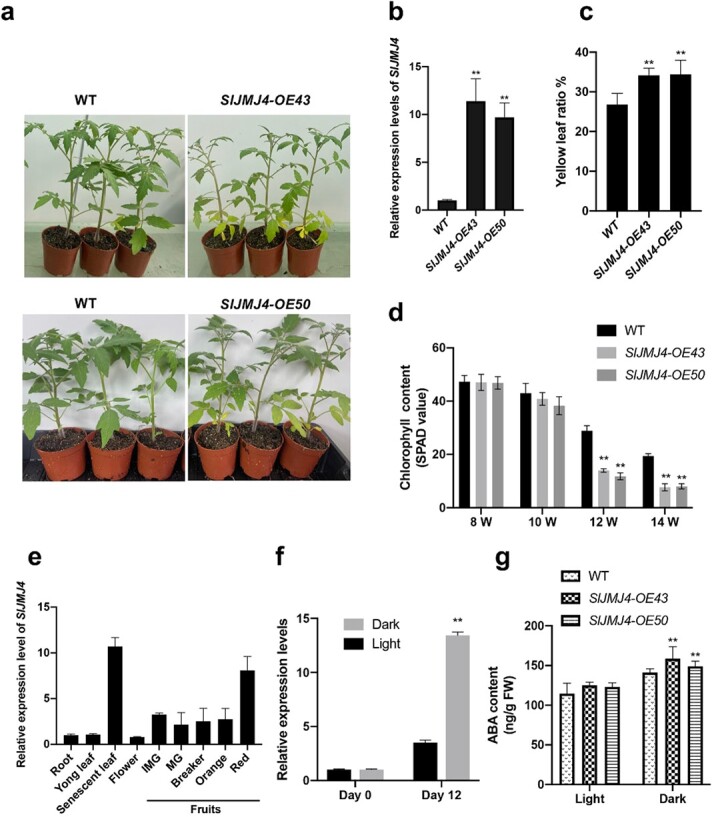
**SlJMJ4 is implicated in the regulation of leaf senescence in tomato**. **a** Phenotype of *SlJMJ4-OE* transgenic plants. Twelve-week-old plants from WT and *SlJMJ4-OE* lines are displayed. **b** The expression of *SlJMJ4* in *SlJMJ4-OE43* and *SlJMJ4-OE50* compared with the WT. Expression was analyzed in one-month-old seedlings. *ACTIN* was used as the reference gene. **c** Yellow leaf ratio of 12-week-old WT, *SlJMJ4-OE43*, and *SlJMJ4-OE50* plants. Leaves with >50% yellowing were counted and divided by the total number of leaves. **d** Chlorophyll content of the bottom third leaf on stems of 8-week-old (8 W), 10-week-old (10 W), 12-week-old (12 W), and 14-week-old (14 W) WT, *SlJMJ4-OE43*, and *SlJMJ4-OE50* plants. Chlorophyll content was measured with a SPAD meter. **e***SlJMJ4* transcript abundance in different tissues of WT tomato plants (cv. Ailsa Craig). IMG, immature green; MG, mature green; Breaker, color break; Orange, turning orange; Red, mature red. **f** Expression of *SlJMJ4* in young detached leaves from the top of the stems of 2-month-old WT plants at 0 d or 12 d after dark incubation. Leaves under daylight conditions were used as the controls. **g** ABA content in leaves from 2-month-old WT and *SlJMJ4-OE* plants at 12 d after dark incubation. In **b**, **c**, **d**, **f**, and **g**, the values are shown as the mean ± SE of three biological replicates (Student’s *t*-test; ^**^P < 0.01).

To explore the function of SlJMJ4 in regulating leaf senescence, we generated *SlJMJ4*-overexpressing lines in the Ailsa Craig background. Eighteen independent transgenic lines were obtained, and two stable lines expressing high levels of *SlJMJ4*, *SlJMJ4-OE43* and *SlJMJ4-OE50*, were selected for further analysis (Fig. 3a, b). Phenotypic analysis showed that *SlJMJ4-*overexpressing plants exhibited obvious premature senescence (Fig. 3a, b). The yellow ratios were significantly higher (Fig. 3c) and the chlorophyll contents were much lower in leaves of 12-week-old *SlJMJ4-OE* plants than in those of the WT (Fig. 3d).

In addition, transcription levels of *SlJMJ4* were analyzed in different tissues, including roots, flowers, young leaves, senescent leaves, and fruits at various ripening stages, as well as under dark conditions. The results showed that *SlJMJ4* was slightly expressed in young leaves, flowers, and roots, moderately expressed in unripe and ripe fruits, and highly expressed in senescent leaves and fruits (Fig. 3e). It appears that the expression of *SlJMJ4* is associated with tissue senescence. Moreover, dark treatment induced the expression of *SlJMJ4* in WT leaves (Fig. 3f), and the ABA content was higher in *SlJMJ4-*overexpressing leaves than in WT leaves under dark induction (Fig. 3g). Collectively, these data suggest that SlJMJ4 plays a positive role in leaf senescence of tomato, which may be related to the phytohormone ABA.

### SlJMJ4 accelerates dark-induced leaf senescence in tomato

**Figure 4:**
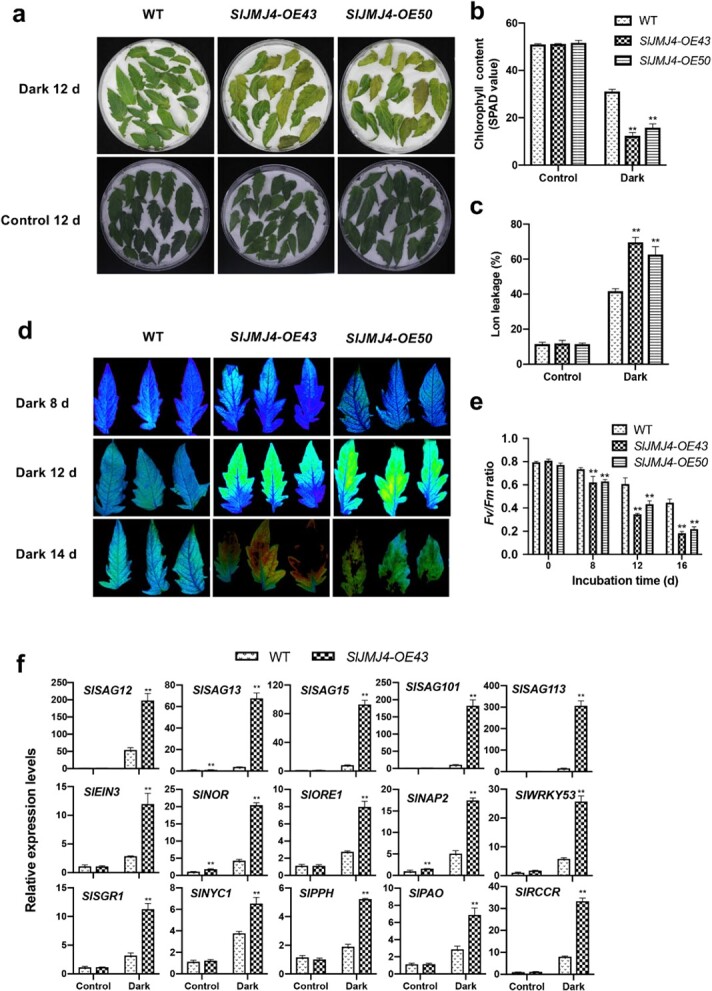
**SlJMJ4 affects dark-induced leaf senescence. a** Phenotypes of detached young leaves from 2-month-old WT, *SlJMJ4-OE43*, and *SlJMJ4-OE5* plants at 12 d after dark incubation at 24°C. For the control, leaves were incubated under continuous white light at 24°C. **b** Chlorophyll contents of WT, *SlJMJ4-OE43*, and *SlJMJ4-OE5* leaves at 12 d after dark incubation or under continuous white light (control). Chlorophyll contents were measured with a SPAD meter. **c** Ion leakage from detached leaves of WT, *SlJMJ4-OE43*, and *SlJMJ4-OE5* plants at 12 d after dark incubation or under continuous white light (control). **d**,**e** Chlorophyll fluorescence images (**d**) and *Fv/Fm* (**e**) of 2-month-old WT, *SlJMJ4-OE43*, and *SlJMJ4-OE50* leaves during dark incubation. **f** Expression levels of *SAGs* and chlorophyll degradation genes in detached leaves from 2-month-old WT and *SlJMJ4-OE43* plants at 12 d after dark incubation or under continuous white light (control). *ACTIN* was used as an internal control for qRT-PCR normalization. In **b**, **c**, **e**, and **f**, the values are shown as the mean ± SE of three biological replicates (Student’s *t*-test; ^**^P < 0.01).

Dark treatment is known to induce senescence of detached leaves in plants, making it a good model for studying leaf senescence [26]. Here, we examined the possible role of SlJMJ4 in regulating dark-induced leaf senescence in tomato. The detached leaves of the *SlJMJ4-OE43* and *SlJMJ4-OE50* lines showed strong senescence phenotypes after 12 d of dark incubation (Fig. 4a), with significantly lower chlorophyll content (Fig. 4b) and higher ion leakage (Fig. 4c) compared with the WT. *Fv*/*Fm* is an important parameter for evaluating leaf senescence. During dark incubation, the *Fv*/*Fm* value of detached WT leaves gradually decreased. However, the value decreased dramatically in *SlJMJ4-OE43* and *SlJMJ4-OE50* leaves (Fig. 4d), indicating that SlJMJ4 promoted the senescence of detached leaves under dark conditions. Moreover, the chlorophyll fluorescence images were consistent with the *Fv/Fm* values in WT and *SlJMJ4-OE* lines during dark incubation (Fig. 4e). Chlorophyll degradation genes, senescence-associated genes, and transcription factor genes such as *NACs* and *WRKY*s are considered to be important positive regulators of leaf senescence. We examined the expression of many genes related to senescence regulation, including *SlSAG12/13/15/101/113*, *SlEIN3*, *SlNOR*, *SlORE1*, *SlNAP2*, and *SlWRKY53.* As shown in Fig. 4f, the expression levels of these genes were markedly upregulated in the *SlJMJ4-OE43* line compared with the WT under dark conditions.

### SlJMJ4 upregulates the expression of *SlSAG12*, *SlSAG113*, *SlNAP2*, and *SlORE1* by removing H3K27me3 under dark conditions

**Figure 5:**
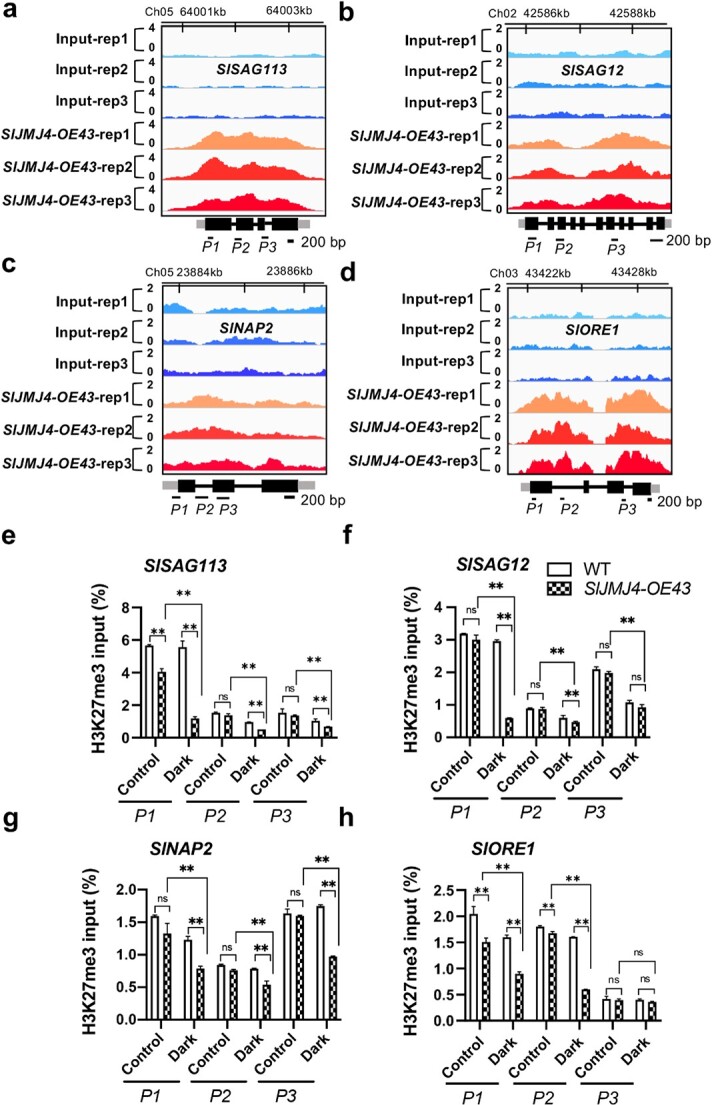
**SlJMJ4 activates the transcription of *SlSAG12*, *SlSAG113*, *SlNAP2*, and *SlORE1* by reducing their H3K27me3 levels. a**–**d** Genome browser visualization of the binding sites of *SlSAG113* (**a**), *SlSAG12* (**b**), *SlNAP2* (**c**), and *SlORE1* (**d**) genes detected by ChIP-seq in the *SlJMJ4-OE* line. Black box, exon; Black line, intron; Grey box, UTR. Black bar, 200 bp. *P1*, *P2*, and *P3* indicate different primer pairs. **e**–**h** ChIP-qPCR analysis of H3K27me3 methylation status at the *SlSAG113* (**e**), *SlSAG12* (**f**), *SlNAP2* (**g**), and *SlORE1* (**h**) loci in detached leaves from WT and *SlJMJ4-OE* plants at 12 d after dark incubation or under continuous white light (control). *ACTIN* was used as an internal reference for ChIP-qPCR. Three biological replicates were used. The data are presented as the mean ± SE of three replicates (Student’s *t*-test, ^**^P < 0.01).

To further investigate the role of SlJMJ4 in leaf senescence, we performed a ChIP-seq analysis to identify the direct targets of SlJMJ4 in the tomato leaves. Leaves from 2-month-old *SlJMJ4-OE* plants were used for immunoprecipitation with anti-GFP antibody and anti-IgG antibody (negative control). A total of 4097 genes, corresponding to 5004 common binding peaks from three biological replicates, were identified (Supplementary Table S1; Supplementary Fig. S2a) and were distributed in different genomic regions, including introns, exons, transcription start sites, promoters, and intergenic areas **(Supplementary Fig. S2b)**. The predominant DNA-binding sites of SlJMJ4 were distributed in intergenic areas and exons **(Supplementary Fig. S2b)**. Meta-gene analysis also showed that SlJMJ4 binding sites were most significantly enriched at transcription end sites **(Supplementary Fig. S2c)**. Motif enrichment analysis showed that the most predominant motif in SlJMJ4-binding sequences was YACGTY (where Y represents A, T, G, or C), which is a binding site of bZIP transcription factors, including HY5, ABI5, TGA4, TGA6, and JUND **(Supplementary Fig. S2d;**Supplementary Table S2). This result implies that SlJMJ4 may be recruited by bZIP transcription factors to regulate gene expression. GO and KEGG pathway enrichment analyses showed that these SlJMJ4-targeted genes were associated with photosynthesis, light harvesting, carboxylic acid metabolism, stress response, and programmed cell death **(Supplementary Figs. S3a and b)**. Gene browser view showed that, among the above senescence-related genes regulated by SlJMJ4 (Fig. 4f), *SlSAG113*, *SlSGA12*, *SlNAP2*, *SlORE1*, *SlSGA15*, *SlEIN3*, *SlWRKY53*, *SlNYC1*, *SlPPH*, and *SlPAO* (Fig. 5a–d; **Supplementary Fig. S4a**) were significantly bound by SlJMJ4, whereas *SlSAG101*, *SlSAG13*, *SlNOR*, *SlSGR*, and *SlRCCR* were not bound by SlJMJ4*,* suggesting that these senescence-related genes may be directly and indirectly regulated by SlJMJ4 **(Supplementary Fig. S4b)**.

The trimethylation of H3K27 is a global epigenetic mark that is usually associated with gene repression [27]. As SlJMJ4 has H3K27me3 demethylase activity, we next examined whether SlJMJ4 regulates the expression of genes related to senescence regulation by H3K27me3 demethylation during dark-induced leaf senescence. We selected four genes related to senescence regulation, *SlSAG12*, *SlSAG113*, *SlNAP2*, and *SlORE1* (Fig. 5a–d), and we compared their H3K27me3 levels in WT and *SlJMJ4-OE43* leaves at 12 d under dark conditions by ChIP-qPCR with an anti-H3K27me3 antibody. As shown in Fig. 5e–h, *SlSAG12*, *SlSAG113*, *SlNAP2*, and *SlORE1* showed no differences in H3K27me3 levels between *SlJMJ4-OE43* and WT lines in the control treatment (continuous light). However, these genes displayed significantly reduced levels of H3K27me3 methylation in *SlJMJ4-OE43* relative to the WT under dark conditions. These results suggest that SlJMJ4 promotes dark-induced leaf senescence, dependent on H3K27me3 demethylation of senescence regulation–related genes.

**Figure 6:**
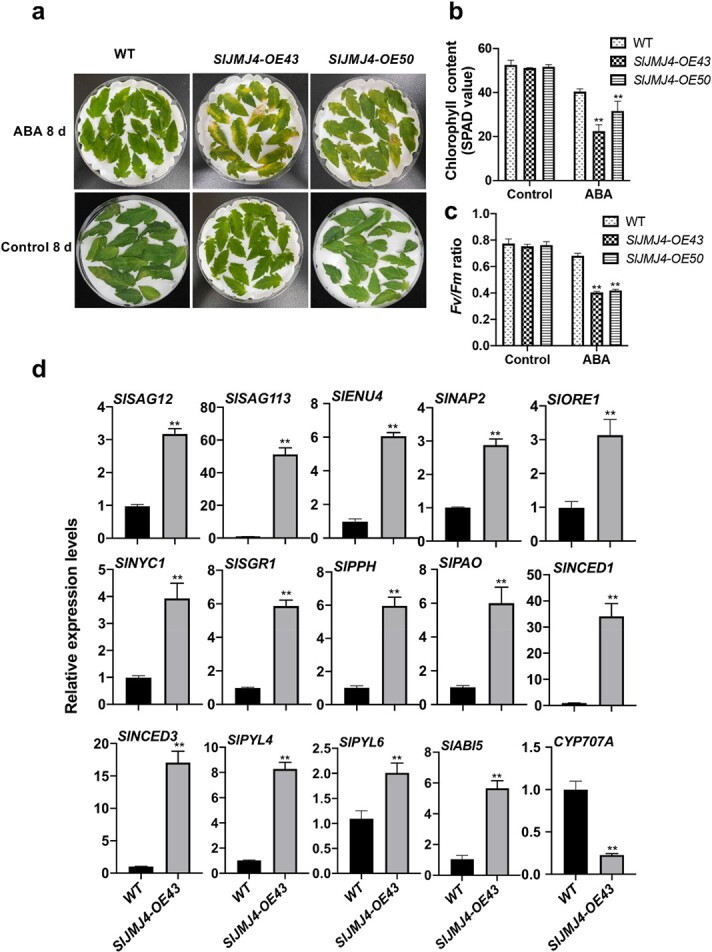
**SlJMJ4 affects ABA-induced leaf senescence. a** Phenotypes of detached leaves from 2-month-old *SlJMJ4-OE* and WT plants at 8 d after ABA treatment. **b** Total chlorophyll content in *SlJMJ4-OE* and WT leaves at 8 d after ABA treatment. **c** Chlorophyll fluorescence *Fv/Fm* values of *SlJMJ4-OE* and WT leaves at 8 d after ABA treatment. **d** Relative expression levels of *SAGs*, chlorophyll degradation genes, and ABA-related genes at 8 d after ABA treatment. *ACTIN* was used as the internal control. The data are presented as the mean ± SE of three replicates (Student’s *t*-test, ^**^P < 0.01).

### SlJMJ4 promotes ABA-induced leaf senescence in tomato

It is well known that ABA is involved in the onset and progression of leaf senescence [28]. Our results showed that dark incubation resulted in higher accumulation of ABA in *SlJMJ4-OE43* and *SlJMJ4-OE50* lines than in WT lines (Fig. 3g). We speculated that SlJMJ4-promoted leaf senescence is related to ABA, and we therefore investigated the role of SlJMJ4 in regulating tomato leaf senescence in response to ABA. Leaves from *SlJMJ4-OE43* and *SlJMJ4-OE50* lines showed a more severe senescence phenotype than the WT and had clearly faded from green to yellow at 8 d after ABA treatment (Fig. 6a). Consistent with their phenotypes, the leaves of WT plants retained higher chlorophyll contents (Fig. 6b) and *Fv/Fm* values (Fig. 6c) than those of *SlJMJ4-OE* plants. qRT-PCR analysis showed that the expression levels of SAGs (*SlSAG12* and *SlSAG113*), chlorophyll degradation–related genes (*SlSGR1*, *SlNYC1*, *SlPHO*, and *SlPAO*), and transcription factor genes (*SlNAP2*, *SlORE1*, and *SlENU4*) were significantly upregulated in the *SlJMJ4-OE43* line compared with the WT (Fig. 6d). Interestingly, the expression levels of many genes related to ABA synthesis (*NCED1* and *NCED3*) and signaling (*SlPYL4*, *SlPYL6*, and *SlABI5*) were also upregulated, whereas expression of the ABA degradation gene *CYP707A* was downregulated in the *SlJMJ4-OE43* line compared with the WT (Fig. 6d). These results showed that SlJMJ4 increased sensitivity to exogenous ABA by upregulating the expression of many genes related to ABA synthesis and signaling, transcription regulation, and senescence. SlJMJ4 may function as an important epigenetic factor involved in ABA-mediated leaf senescence.

### SlJMJ4 upregulates the transcription of *SlSAG113*, *SlSAG12*, *SlNAP2*, *SlORE1*, *SlNCED3*, and *SlABI5* by removal of H3K27me3 in response to ABA

To further elucidate the involvement of SlJMJ4 in the regulation of tomato leaf senescence in response to ABA, we investigated the binding and K3K27me3 levels of many target genes in the WT and *SlJMJ4-OE* after ABA treatment. In addition to *SlSAG12*, *SlSAG113*, *SlNAP2*, and *SlORE1*, we found that two genes related to ABA synthesis and signaling, *SlNCED3* and *SlABI5*, were also the direct targets of SlJMJ4 (Fig. 7a–b; Supplementary Table S1). Three different loci (*P1*, *P2*, and *P3*) were selected with the Integrative Genomics Viewer (IGV) to explore the binding of SlJMJ4 to the loci of *SlSAG12*, *SlSAG113*, *SlNAP2*, *SlORE1* (Fig. 5a–d), *SlNCED3*, and *SlABI5* (Fig. 7a–b) by ChIP-qPCR. The results confirmed that SlJMJ4 bound directly to the loci of these genes. Moreover, the binding of SlJMJ4 at the loci of *SlSAG12*, *SlSAG113*, *SlNAP2*, *SlORE1*, *SlNCED3*, and *SlABI5* were intensified in *SlJMJ4-OE43* leaves after ABA treatment, indicating that ABA treatment resulted in increased binding of SlJMJ4 at target gene loci (Fig. 7c–h). In addition, we also analyzed H3K27me3 methylation status at the *SlSAG12*, *SlSAG113*, *SlNAP2*, *SlORE1*, *NCED3*, and *ABI5* loci. The H3K27me3 levels of these genes were significantly lower in *SlJMJ4-OE43* leaves than in WT leaves at 8 d after ABA treatment (Fig. 7i–n). Taken together, these results indicated that SlJMJ4 increased sensitivity to ABA by binding to many key genes related to ABA synthesis and signaling, transcription regulation, and senescence, thereby promoting their H3K27me3 demethylation. More importantly, our research established a close relationship between SlJMJ4-mediated H3K27me3 demethylation and the ABA response for regulating leaf senescence in tomato.

**Figure 7:**
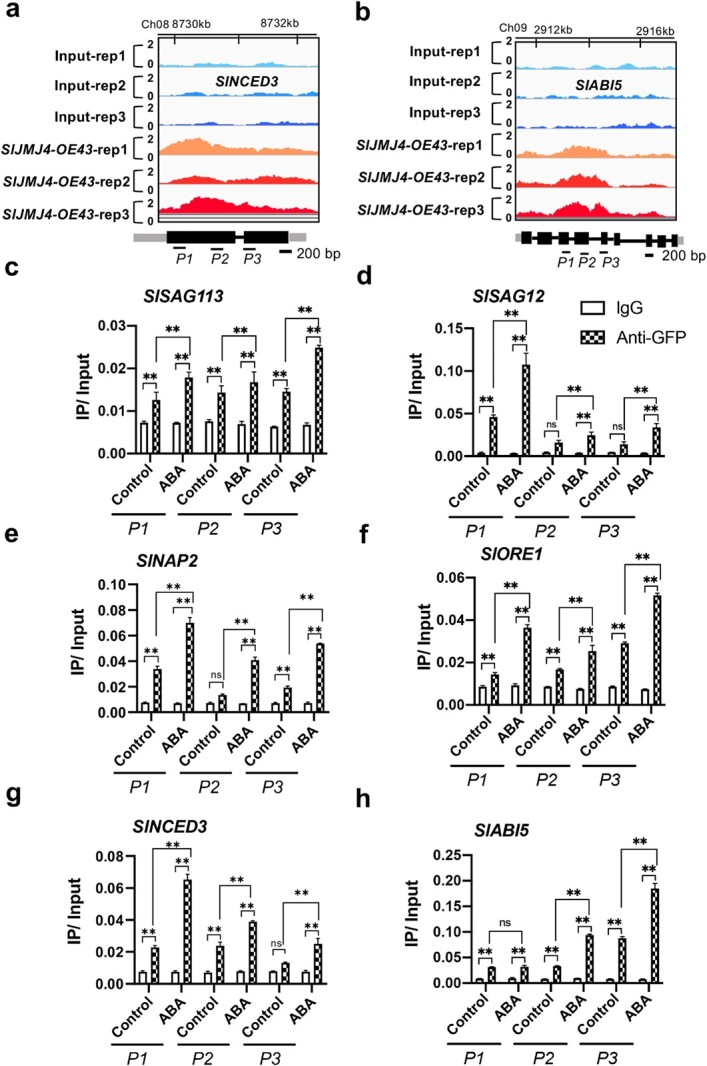
**ChIP-qPCR analysis of the binding and H3K27me3 methylation status of SlJMJ4 at its target gene in response to ABA. a**,**b** Genome browser visualization of the binding sites of *SlABI5* (**a**) and *SlNCED3* (**b**) genes detected by ChIP-seq in the *SlJMJ4-OE* line. Black box, exon; Black line, intron; Grey box, UTR. Black bar, 200 bp. *P1*, *P2,* and *P3* indicate different primer pairs. **c**–**h** ChIP-qPCR analysis of the binding of SlJMJ4 at the loci of *SlSAG113* (**c**), *SlSAG12* (**d**), *SlNAP2* (**e**), *SlORE1* (**f**), *SlNCED3* (**g**), and *SlABI5* (**h)** in detached leaves from 2-month-old *SlJMJ4-OE* and WT plants at 8 d after ABA treatment. An anti-GFP antibody was used for immunoprecipitation, and IgG was used as the negative control. **i**–**n** ChIP-qPCR analysis of H3K27me3 methylation status at the loci of *SlSAG113* (**i**), *SlSAG12* (**j**), *SlNAP2* (**k**), *SlORE1* (**l**), *SlABI5* (**m**), and *SlNCED3* (**n**) in detached leaves from 2-month-old *SlJMJ4-OE* and WT plants at 8 d after ABA treatment. *ACTIN* was used as an internal reference for ChIP-qPCR. Three biological replicates were used. The data are presented as the mean ± SE of three replicates (Student’s *t*-test, ^**^P < 0.01).

**Figure 7:**
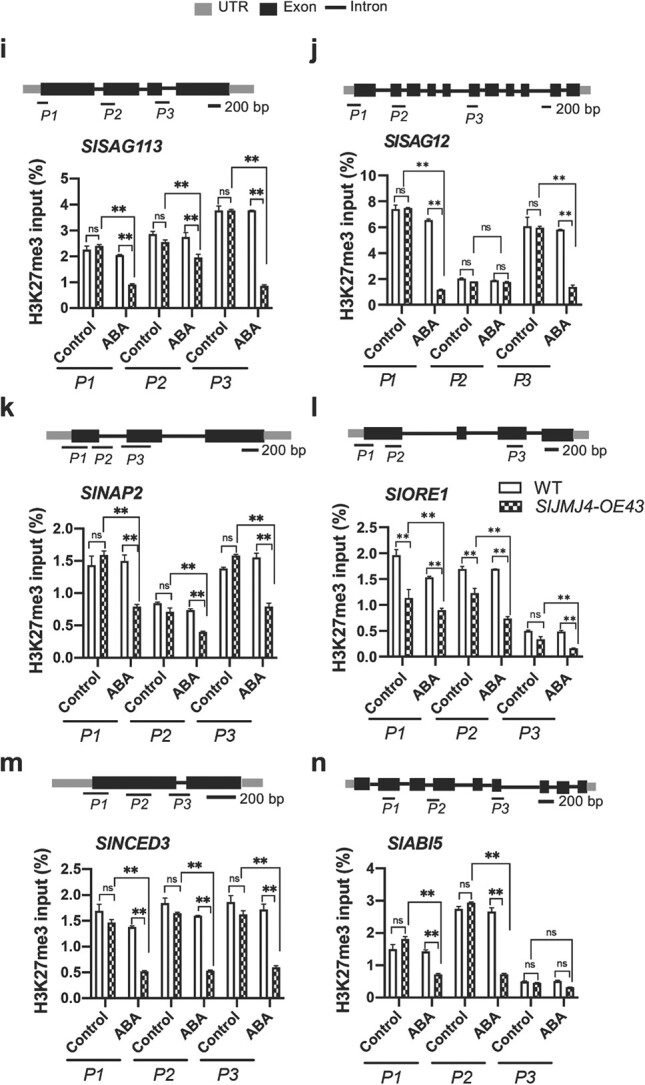
(Continued)

## Discussion

Histone demethylases play important roles in regulating histone methylation level and gene expression in plants [15]. In the model plants Arabidopsis and rice, the involvement of histone demethylase in regulating physiological processes has been extensively elucidated. However, relatively few histone demethylases have been characterized in other plant species. Here, we characterized the expression, subcellular localization, and histone demethylase activity and specificity of SlJMJ4 and further elucidated its possible role in the regulation of tomato leaf senescence.

### SlJMJ4 is an H3K27 demethylase

Histone lysine methylation is an important epigenetic mark that is crucial for regulating diverse biological processes [29]. Histone lysine methylation mainly occurs at K4, K9, K27, and K36 in histone H3, and it is dynamically regulated by histone demethylases and methyltransferases [15]. The involvement of H3K9 and H3K4 demethylases in biological processes has been widely reported in Arabidopsis and rice [15], whereas relatively few H3K27 demethylases have been characterized. Arabidopsis REF6 (JMJ12) was the first H3K27me2/3 demethylase reported in plants [30]. Subsequently, ELF6 (JMJ11), JMJ13, and JMJ30 were also confirmed as H3K27 demethylases [31–33]. In the present study, we identified SlJMJ4, which was classified into the KDM4/JHDM3 subgroup, as an H3K27 demethylase from tomato. Enzymatic activity analyses *in vitro* demonstrated that SlJMJ4 specifically demethylates histone H3K27me2/3 (Fig. 2b). Moreover, overexpression of *SlJMJ4* in tomato plants reduced the global levels of H3K27me2/3 (Fig. 2a). Therefore, SlJMJ4 acts as an H3K27me2/3 demethylase in tomato.

### SlJMJ4 promotes the transcription of functional and regulatory genes related to senescence by reducing their H3K27me3 levels during dark-induced leaf senescence

Among the different epigenetic mechanisms, histone methylation modifications have been extensively investigated and characterized. In most eukaryotic genomes, a large proportion of chromatin is enriched with H3K27me3 [33]. There is mounting evidence that histone lysine methylation modifications regulated by H3K27 demethylases are involved in plant developmental and physiological processes, especially the floral transition. The H3K27me3 demethylases REF6 [34] and ELF6 [16, 35] directly regulate the central flowering regulators FLOWERING LOCUS T (FT) and FLOWERING LOCUS C (FLC) and control the transition from vegetative growth to flowering in Arabidopsis. The *elf6* and *ref6* mutants display early and late flowering phenotypes, respectively. Similarly, the mediation of H3K27 demethylation at the FLC locus by JMJ30 and its homolog JMJ32 contributes to the thermal stability of flowering at elevated temperatures [32]. In addition, the H3K27me3 demethylase JMJ705 is recruited by WUSCHEL-related homeobox 11 to promote gene expression during shoot growth in rice [36].

Currently, there is little information about the role of H3K27 demethylase in regulating plant senescence. The H3K27me3 demethylase REF6 is suggested to promote leaf senescence by activating numerous functional and regulatory genes related to senescence in Arabidopsis [21]. In this study, we generated *SlJMJ4-*overexpressing transgenic tomato plants and found that they had an obvious premature senescence phenotype, with earlier de-greening, lower chlorophyll content, and higher yellowing ratio than the WT at 12 weeks (Fig. 3). Moreover, when subjected to dark conditions for 12 d, *SlJMJ4-*overexpressing leaves showed lower chlorophyll contents, lower chlorophyll fluorescence, and higher ion leakage compared with the WT. These results indicate that SlJMJ4 promotes leaf senescence in an age-dependent manner and under dark conditions. SlJMJ4 had high homology with AtJMJ13 and OsJMJ706 (Fig. S1b). AtJMJ13 is an H3K27me3 demethylase and a flowering inhibitor [24], whereas OsJMJ706 has H3K9me2/3 demethylase activity and regulates rice flowering [25]. Neither of these histone demethylases has been reported to regulate leaf senescence. We have constructed *SlJMJ4*, *SlJMJ6* [18], and *SlJMJ7* (data not shown) overexpression lines, and only the *SlJMJ4* overexpression line exhibited the premature leaf senescence phenotype. SlJMJ6 delays tomato fruit ripening, and SlJMJ7 promotes tomato fruit ripening, but neither promote the premature leaf senescence phenotype. It appears that different SlJMJs may have specific biological functions.

Senescence begins with chlorophyll degradation, involving a number of chlorophyll degradation–related genes, SAGs, and regulators [10]. A set of key genes, including pheophytin pheophorbide hydrolase (*PPH*), STAY-GREEN 1 (*SGR1*), NON-YELLOWING 1 (*NYE1*), chlorophyll catabolite reductase (*RCCR*), and pheophorbide a oxygenase (*PAO*), are involved in this process [10]. In addition, as revealed by genomic, genetic, metabolomic, proteomic, and transcriptomic research, leaf senescence is dynamically regulated by numerous SAGs [37]. Furthermore, several transcription factors, including OsNAP [38], ORE1 [3], AtNAP2 [2, 39], and WRKY53 [40], play important roles in regulating chlorophyll degradation–related genes and SAGs. In this study, the overexpression of *SlJMJ4* in tomato plants resulted in the upregulated expression of a large number of senescence-related regulatory genes under dark conditions, including chlorophyll degradation–related genes (*SlSGR1*, *SlNYC1*, *SlPPH*, *SlPAO*, and *SlRCCR*), transcription factor genes (*SlEIN3*, *SlNOR*, *SlORE1*, *SlNAP2*, and *SlWRKY53*) and *SAGs* (*SlSAG12/13/15/101/113*) (Fig. 4). These results further indicated that the histone H3K27 demethylase SlJMJ4 positively regulates tomato leaf senescence by activating numerous functional and regulatory genes related to senescence.

Chromatin status plays an important role in transcriptional regulation and other chromatin-based nuclear processes. Histone lysine methylation affects gene expression by altering chromatin status. In general, H3K27 and H3K9 histone methylation are related to inactive chromatin and gene silencing, whereas H3K4 and H3K36 methylation are associated with gene activation [41]. Studies have shown that Arabidopsis leaf senescence involves dynamic changes in H3K4me3 [42] and H3K27me3 [43]. Recently, Liu et al. [22] reported that JMJ16 negatively regulates Arabidopsis leaf senescence by suppressing the expression of *WRKY53* and *SAG201*, two positive regulators. However, REF6 has the opposite effect [21]. The down- and upregulation of senescence-related genes by JMJ6 and REF6 are associated with the removal of H3K4 and H3K27 methylation, respectively [21, 22]. In the present study, in response to dark conditions, the levels of H3K27me3 at the loci of *SlSAG113*, *SlSAG12*, *SlNAP2*, and *SlORE1*, four positive regulators of leaf senescence, were significantly downregulated in *SlJMJ4-*overexpressing lines compared with the WT. Considering that H3K27me3 is a repressive mark, the decreased levels of H3K27me3 were consistent with the upregulated expression of these genes in *SlJMJ4-*overexpressing lines relative to the WT (Fig. 5). Taken together, these results indicate that SlJMJ4-mediated demethylation of H3K27m3 at senescence-related gene loci is important for the regulation of tomato leaf senescence.

### SlJMJ4 affects ABA-induced leaf senescence by reducing H3K27me3 levels of ABA-related genes to regulate their transcription

Senescence is triggered by various endogenous and environmental signals. The plant hormone ABA plays an important role in the onset and progression of leaf senescence [28]. Under external stimuli, ABA is rapidly synthesized and then sensed by ABA receptors, ultimately activating a cascade of transcription factors [40]. Previous work suggests that ABA stimulates a set of *SAGs* by transcriptional regulation in ABA signaling pathways to drive leaf senescence [44]. However, the mechanism by which ABA induces *SAG* expression at the onset of leaf senescence still remains to be elucidated.

Recent studies have revealed that epigenetic modification is implicated in leaf senescence in response to ABA. ABA induces leaf senescence by decreasing H3K27me3 of *SAGs* [45]. *ABREs* (ABA-responsive elements) are subjected to H3K27me3 modification via polycomb repressive complex 2 (PRC2) [46], as well as the H3K27 trimethyltransferases CLF and SWN [47]. The *clf swn* double mutants are hypersensitive to ABA, with decreased H3K27me3 levels at *SAG* gene loci compared with WT plants [48]. More recently, Wang et al. [49] reported that the H3K4 demethylase JMJ17 is recruited to *ABI5* chromatin by interacting with WRKY40 upon ABA exposure. In this study, SlJMJ4 increased the sensitivity of tomato leaves to ABA, with accelerated leaf senescence in *SlJMJ4-OE* leaves compared with WT leaves (Fig. 6). ABA is synthesized from β-carotene and 9-cis-epoxycarotenoid dioxygenase, and NCED is a rate-limiting enzyme for ABA synthesis [44]. Upon ABA treatment, the H3K27me3 level at the *SlNCED3* locus was greatly decreased in *SlJMJ4-OE43* plants compared with the WT (Fig. 7), consistent with upregulated *SlNCED3* expression. ABI5 is a downstream transcription factor in the ABA signaling pathway and plays a key positive regulatory role in the ABA response [49]. In Arabidopsis, ABI5 binds to the promoter of *ORE1* and promotes its expression and leaf senescence [50]. Overexpression of *ORE1* triggers early senescence by controlling downstream *SAGs*, whereas its inhibition delays senescence in Arabidopsis [51]. In tomato, SlABI5 regulates *SlSGRL* expression by directly binding to the ABRE cis-element to promote chlorophyll degradation [44]. In addition, SlNAP2 binds directly to the promoters of *SlSAG113*, *SlSGR1*, *SlPAO*, and other downstream targets to activate their expression and promote leaf senescence [52]. In the present study, in response to ABA, SlJMJ4 upregulated the expression of *SlABI5*, *SlNAP2*, *SlORE1*, *SlSAG113*, and *SlSAG12* by removing the H3K27me3 at their loci (Figs. 6 and 7), thereby activating the ABA signaling pathway.

**Figure 8:**
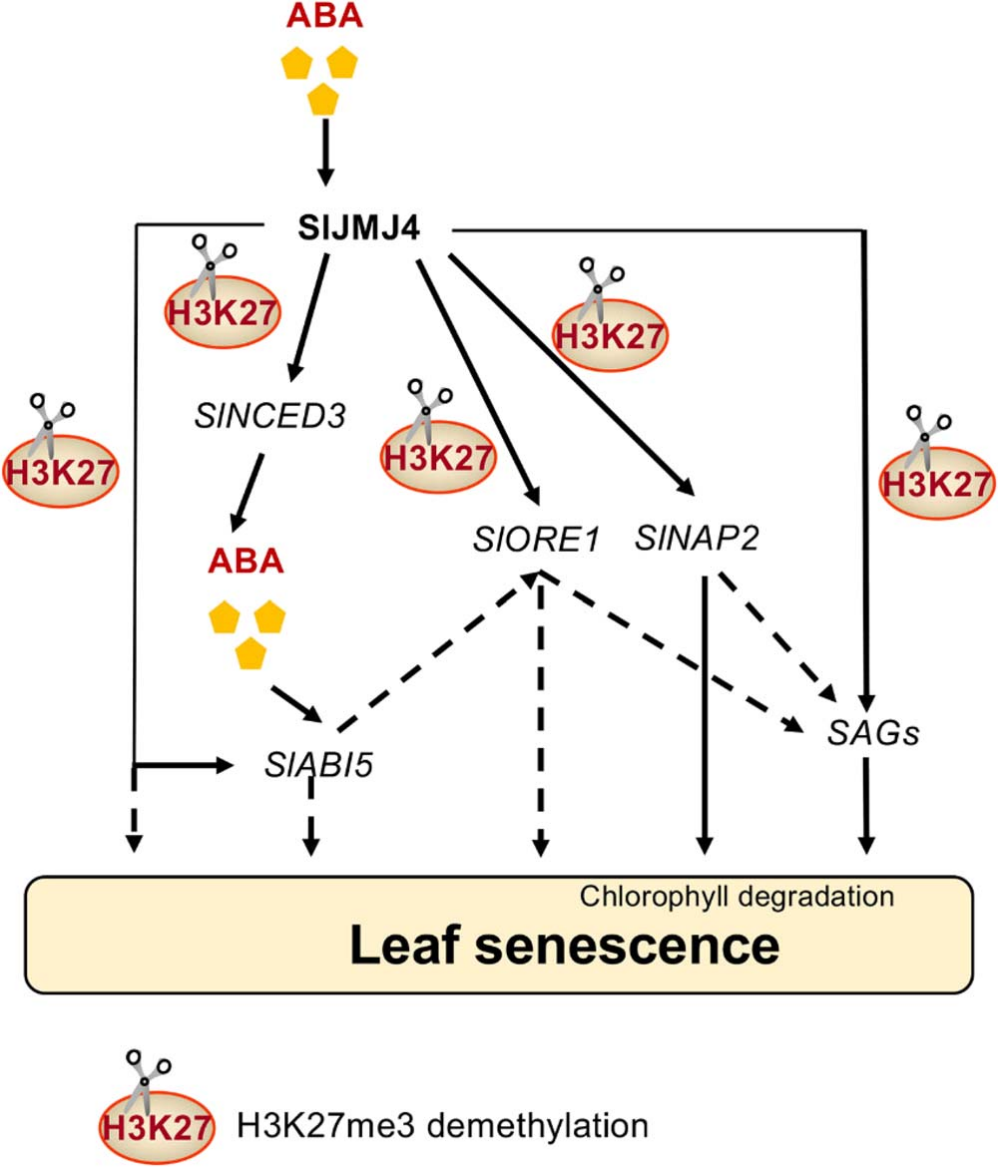
**A proposed model to explain the involvement of SlMJM4 in regulating tomato leaf senescence in response to ABA.** After ABA treatment, H3K27me3 levels of *SlNCED3* genes decreased in *SlJMJ4-OE43* lines, thus promoting ABA synthesis. The H3K27 demethylation of *SlABI5* by SlJMJ4 is induced by ABA, which promotes ABA signal transduction and activates the expression of *SlABI5* and downstream *SlORE1* and *SAG* genes, thereby stimulating leaf senescence. Moreover, SlJMJ4 directly upregulates the expression of *SlORE1*, *SlNAP2*, *SlSAG113*, and *SlSAG12* genes via H3K27me3 demethylation. In summary, SlJMJ4 is involved in the ABA-induced senescence of tomato leaves by activating the transcription of many genes associated with ABA synthesis and signal transduction via removal of H3K27me3. The solid arrows indicate confirmed processes, whereas the dotted arrows indicate speculation based on our work and previous studies.

Based on the above results, we proposed a model to explain the involvement of SlMJM4 in regulating tomato leaf senescence in response to ABA (Fig. 8). After ABA treatment, H3K27me3 levels of *SlNCED3* genes decreased in *SlJMJ4-OE43* lines, thus promoting ABA synthesis. The H3K27 demethylation of *SlABI5* by SlJMJ4 is induced by ABA, which promotes ABA signal transduction and activates the expression of *SlABI5* and downstream *SlORE1* and *SAG* genes, thereby stimulating leaf senescence. Moreover, SlJMJ4 directly upregulates the expression of *SlORE1*, *SlNAP2*, *SlSAG113*, and *SlSAG12* genes via H3K27me3 demethylation. In summary, SlJMJ4 is involved in the ABA-induced senescence of tomato leaves by binding to many genes related to ABA synthesis and signal transduction, promoting their H3K4me3 demethylation.

Histone demethylases act by regulating gene transcription and chromatin structure. However, it is not clear how the histone demethylases recognize and bind to specific genomic sites. Cui et al. [30] and Li et al. [14] reported that AtREF6/AtJMJ12 may directly bind to their targets and remove H3K27me3 by four Cys2-His2 zinc fingers (ZnF-C2H2) that directly recognize a CTCTGYTY motif. AtJMJ14 is recruited to targets via interaction with the transcription factors NAC050/052 through its FYRN and FYRC domains [53]. In this study, SlJMJ4 contained no ZnF-C2H2 domains. However, SlJMJ4 is associated with a number of loci harboring the binding motifs of bZIP transcription factors, including ABI5, HY5, ABF5, TGA4, TGA6, TAG10, and JUND, implying that SlJMJ4 may be recruited by transcription factors and bind indirectly to its targets. Further biochemical and molecular experiments are required to illustrate the underlying mechanism.

In conclusion, we identified a tomato H3K27me2/3 demethylase, SlJMJ4, which plays a positive role in leaf senescence in tomato. Under dark conditions or in response to ABA, overexpression of *SlJMJ4* results in decreased H3K27me3 levels of many genes related to transcription regulation, chlorophyll degradation, and ABA synthesis and signaling, activating their expression and accelerating leaf senescence in tomato. These results revealed a relationship between SlJMJ4-mediated H3K27me3 demethylation and the ABA response for regulating leaf senescence in tomato.

## Materials and methods

### Plant material and growth conditions

Tomato (*Solanum lycopersicum* L. cv. Ailsa Craig) was used as the wild type (WT), and transgenic lines were generated in the WT background. The WT and transgenic lines were grown under long-day conditions (16 h light/8 h dark) with 65–70% relative humidity at 24°C.

### Sequence analysis

Sequence analyses of SlJMJs used the Conserved Domain Database (CDD) [54] (https://www.ncbi.nlm.nih.gov/Structure/cdd/wrpsb.cgi) and the Simple Modular Architecture Tool (SMART) (http://smart.embl-heidelberg.de) [55]. Protein alignment was performed using phmmer at Ensembl Plants (http://plants.ensembl.org/hmmer/index.html) [56] and Clustal Omega (http://www.ebi.ac.uk/Tools/msa/clustalo) [57]. The Sequence Manipulation Suite (http://www.bioinformatics.org/sms2/color_align_cons.html) [58] was used to highlight conserved regions within the alignments.

### Subcellular localization

An SlJMJ4-pSAT6-GFP fusion vector was constructed, and the fusion vector or a control vector was co-transformed with an mCherry vector into Arabidopsis protoplasts as previously described by Yoo et al. [59]. After 2 d of incubation, GFP and mCherry fluorescence were observed under a fluorescence microscope (Leica SP8 STED 3X) and detected at 488 and 590 nm, respectively.

### Vector construction and transgenic plant generation

The full-length cDNA of *SlJMJ7* was subcloned into the pBI121-GFP vector using the In-Fusion HD Cloning Kit (TaKaRa, Japan). Transgenic plant generation of *SlJMJ4* overexpression lines was carried out as previously described by Li et al. [18]. In brief, the constructs were transformed into *Agrobacterium tumefaciens* GV3101, which was subsequently used to infiltrate young cotyledon sections at 23°C under a 16-h light/8-h dark photoperiod. Transformants were selected based on their resistance to kanamycin. T2 homozygous progeny were used for phenotypic and molecular characterization.

### Quantitative RT-PCR analysis (qRT-PCR)

The RNeasy Plant Mini Kit (Qiagen) was used to extract total tissue RNA in accordance with the manufacturer’s instructions. qRT-PCR was performed on an ABI PRISM 7900HT sequence detection system (Applied Biosystems) using SYBR Green (Applied Biosystems). *ACTIN* (*Solyc03g078400*) was used as the reference gene for data normalization. Gene-specific primers for qRT-PCR are listed in Supplementary Table S3.

### Histone H3K27 demethylation assay *in vitro* and *in vivo*

For *in vitro* demethylation assays, SlJMJ4-GST fusion proteins were purified using glutathione sepharose 4B (GE Healthcare). Afterwards, histone demethylase activity was analyzed as previously described [18]. In brief, the purified GST-tagged SlJMJ14 (4.0 μg) was incubated with calf thymus histones (Sigma) in a reaction buffer containing 150 mM NaCl, 80 μM Fe(NH_4_)_2_(SO_4_)_2_, 50 mM Tris–HCl (pH 7.0), 1 mM α-KG, and 2 mM ascorbic acid for 6 h at 37°C. The reaction was terminated with 10 μM EDTA and subjected to western blotting analysis. For *in vitro* demethylation assays, histone proteins were extracted from 2-month-old leaves of *SlJMJ4-OE* and WT plants with the EpiQuik Total Histone Extraction Kit (Epigentek, Farmingdale, NY, USA) and analyzed by western blotting. The antibodies used in this experiment were from Abcam: H3K4me1 (ab176877, 1:3000 dilution), H3K4me2 (ab11946, 1:3000 dilution), H3K4me3 (ab8580, 1:3000 dilution), H3K9me1 (ab9045, 1:3000 dilution), H3K9me2 (ab1220, 1:1000 dilution), H3K9me3 (ab8898, 1:1000 dilution), H3K27me1 (ab115068, 1:3000 dilution), H3K27me2 (ab24684, 1:3000 dilution), H3K27me3 (ab6002, 1:3000 dilution), H3K36me1 (ab176920, 1:3000 dilution), H3K36me2 (ab176921, 1:3000 dilution), H3K36me3 (ab9050, 1:3000 dilution), and H3 (ab1791, 1:5000). H3 was used as a loading control.

### Chromatin immunoprecipitation (ChIP) and ChIP-seq analysis

ChIP was performed as previously described with slight modifications [60]. Leaves from 2-month-old tomato plants were cross-linked with 1% formaldehyde. The extracted chromatin was sheared to lengths of 100–300 bp by sonication, then immunoprecipitated using anti-GFP antibody (Ab290, Abcam, 1:200 dilution). The precipitated DNA was recovered and sequenced using the NovaSeq PE150 platform. Trimmomatic software was used to eliminate adapters and low-quality reads. The clean reads were then mapped to the tomato reference genome (SL3.0). The mapped reads were processed with MACS2 software using default parameters to identify enriched peaks for each replicate of ChIP-seq data. The findMotifsGenome.pl tool in HOMER was used for motif analysis.

### Dark and ABA treatments

Senescence experiments on detached leaves were performed using the top third and fourth leaves from stems of 2-month-old plants. For dark treatment, detached leaves were incubated on moist filter papers in the dark at 24°C for 14 d. Leaves subjected to continuous white light incubation at 24°C served as the controls. For ABA treatment, detached leaves from WT and *SlJMJ4-OE* plants were treated with 50 μM ABA or solvent methanol as the control [52], then placed on moist filter papers in a petri dish (150 mm diameter) for 8 d under constant light at 24°C.

### Measurement of senescence parameters

The total chlorophyll content was measured with a SPAD-502 chlorophyll meter (Konica-Minolta). Chlorophyll fluorescence was determined using a modulated Fluorometer (OS-500, Opti-Sciences) and expressed as *Fv/Fm* [61]. ABA was obtained by the method of Forcat et al. [62] and measured using tandem mass spectrometry (MS/MS, Applied Biosystems 6500 Quadrupole Trap) and high performance liquid chromatography (HPLC, Agilent 1290). Ion leakage was determined as described previously [63].

### ChIP-qPCR

Tomato leaves subjected to dark treatment or ABA treatment were used for ChIP as mentioned above. For ChIP-qPCR, 95% of the chromatin was used for immunoprecipitation with anti-H3K27me3 (Millipore 07-473, 1:200 dilution), IgG (Millipore, 12-370, 1:500 dilution), and anti-GFP (Abcam, ab290, 1:200 dilution) antibodies, and the remaining 5% was used as the input control. The input and precipitated DNA were subjected to qRT-PCR. Gene-specific primers for qRT-PCR are listed in Supplementary Table S3.

### Statistical analysis

Data are expressed as the mean ± standard error (SE). Differences among treatments were determined by ANOVA followed by Student’s *t*-test.

## Supplementary Material

Web_Material_uhab077

## Data Availability

Both processed and raw ChIP-seq data are stored at the NCBI GEO repository (http://www.ncbi.nlm.nih.gov/geo; accession number GSE177487). The antibodies used in this article are commercial antibodies, and antibody information (e.g. manufacturer, including sufficient address details to enable contact) are provided in the Materials and Methods. The supplementary data that support the findings of this study are openly available in the figshare public repository at https://figshare.com/s/ec8c9a7667870413e221.
