# Correction to: The histone H3K27 demethylase SlJMJ4 promotes dark- and ABA-induced leaf senescence in tomato

**DOI:** 10.1093/hr/uhaf046

**Published:** 2025-02-27

This is a correction to: Xiaochun Ding, Dandan Zhang, Dachuan Gu, Zhiwei Li, Hanzhi Liang, Hong Zhu, Yueming Jiang, Xuewu Duan, The histone H3K27 demethylase SlJMJ4 promotes dark- and ABA-induced leaf senescence in tomato, *Horticulture Research*, Volume 9, 2022, 10.1093/hr/uhab077.

In the originally published version of the manuscript there was an error in Figure 4d.

During the manuscript preparation process, two images were duplicated due to an oversight. These are the **Dark 14d fluorescence image of WT** (colum 1, panel 3) and the **Dark 8d fluorescence image of SlJMJ4-OE50** (column 3, panel 1).

Panel d in Figure 4 should read:

The corrections do not affect the scientific integrity of the study’s conclusion. The authors would like to apologize for this error.

The emendations are outlined only in this notice to preserve the version of record.



