# Supplementary material for: The histone H3K27 demethylase SlJMJ4 promotes dark- and ABA-induced leaf senescence in tomato
Source: Hortic Res. 2022 Jan 19;9:uhab077. doi: 10.1093/hr/uhab077 (PMC8973004; doi:10.1093/hr/uhab077)
Supplement: Web_Material_uhab077 [file web_material_uhab077.zip › Supplementary Figures 1-4.docx]

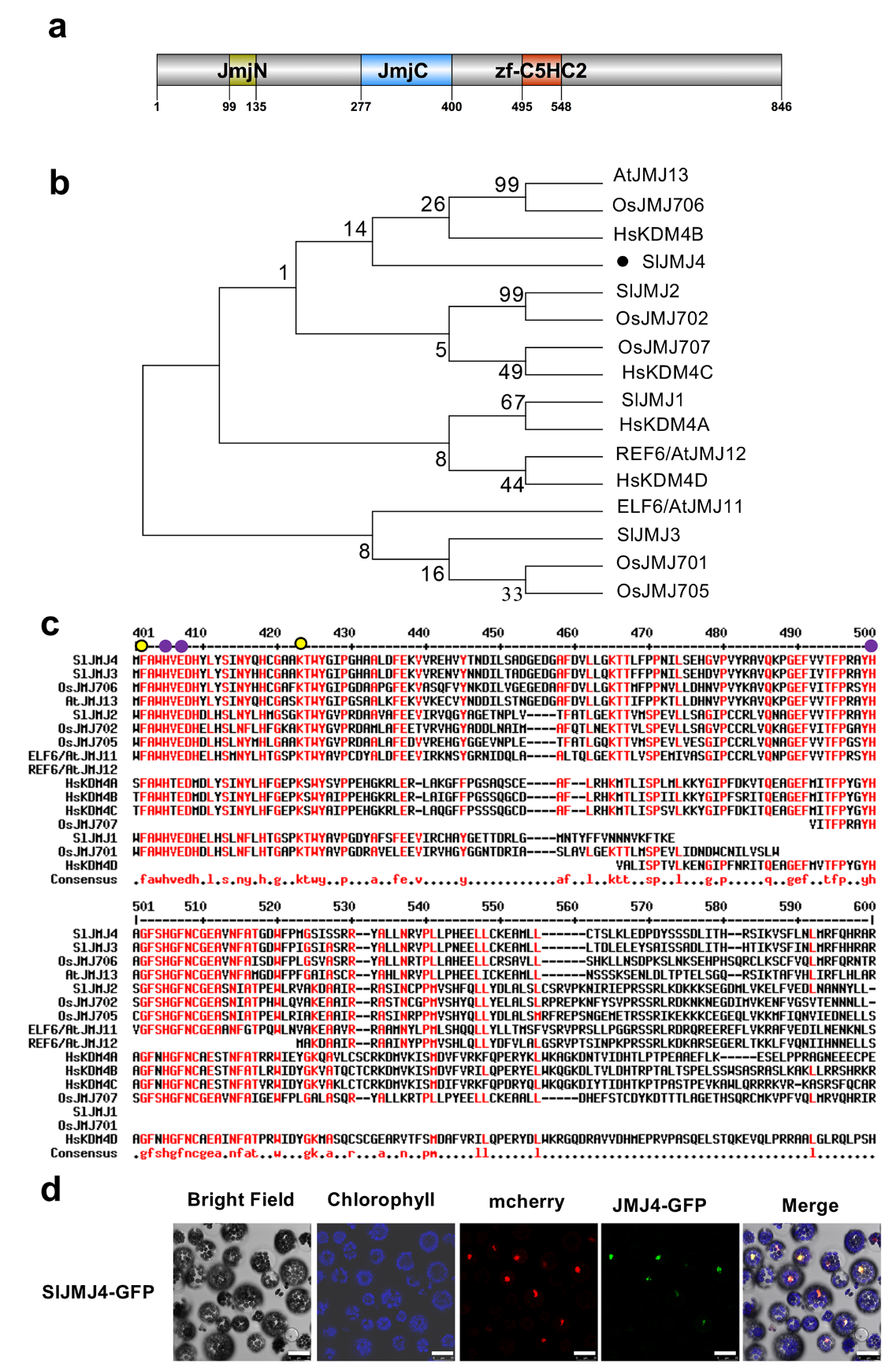


**Fig. S1. Bioinformatic analysis and subcellular localization of SlJMJJ4.** **(a)** Schematic domain structure of SlJMJ4. The protein form of SlJMJ4 contains the JmjN domain (dark yellow color), JmjC domain (blue color), and zinc finger domain (orange color). **(b)** Phylogenetic analysis of SlJMJ4 with other KDM4/ JHDM3 subfamily of JmjC domain-containing proteins. The phylogenetic tree was created with neighbor-joining test with MEGA7.0 program. **(c)** Multiple alignment of SlJMJ4 protein with other JMJ members. The conserved residues compatible with the demethylation activity within the Fe (II) binding site are highlighted in purple circle and those in the α-KG binding site are emphasized in yellow circle. The following proteins were used for analysis: SlJMJ4 (Solyc08g076390), SlJMJ1 (Solyc04g028580), SlJMJ2 (Solyc03g111590), SlJMJ3 (Solyc08g005240), ELF6/JMJ11 (AT5G04240), REF6/JMJ12 (AT3G48430), OsJMJ701 (Os03t0151300), OsJMJ702 (Os12t0279100), OsJMJ705 (Os01t0907400), OsJMJ706 (Os10t0577600), OsJMJ707 (Os02t0696700), HsKDM4A (O75164), HsKDM4B (O94953), HsKDM4C (Q9H3R0) and HsKDM4D (Q6B0I6). **(d)** Subcellular localization of SlJMJ4. SlJMJ4 protein fused to green fluorescent protein (GFP) was transiently expressed in Arabidopsis protoplast. Autofluorescence of plastids, blue; and nuclear localization signal of mCherry, red. Scale bar: 25 μm.


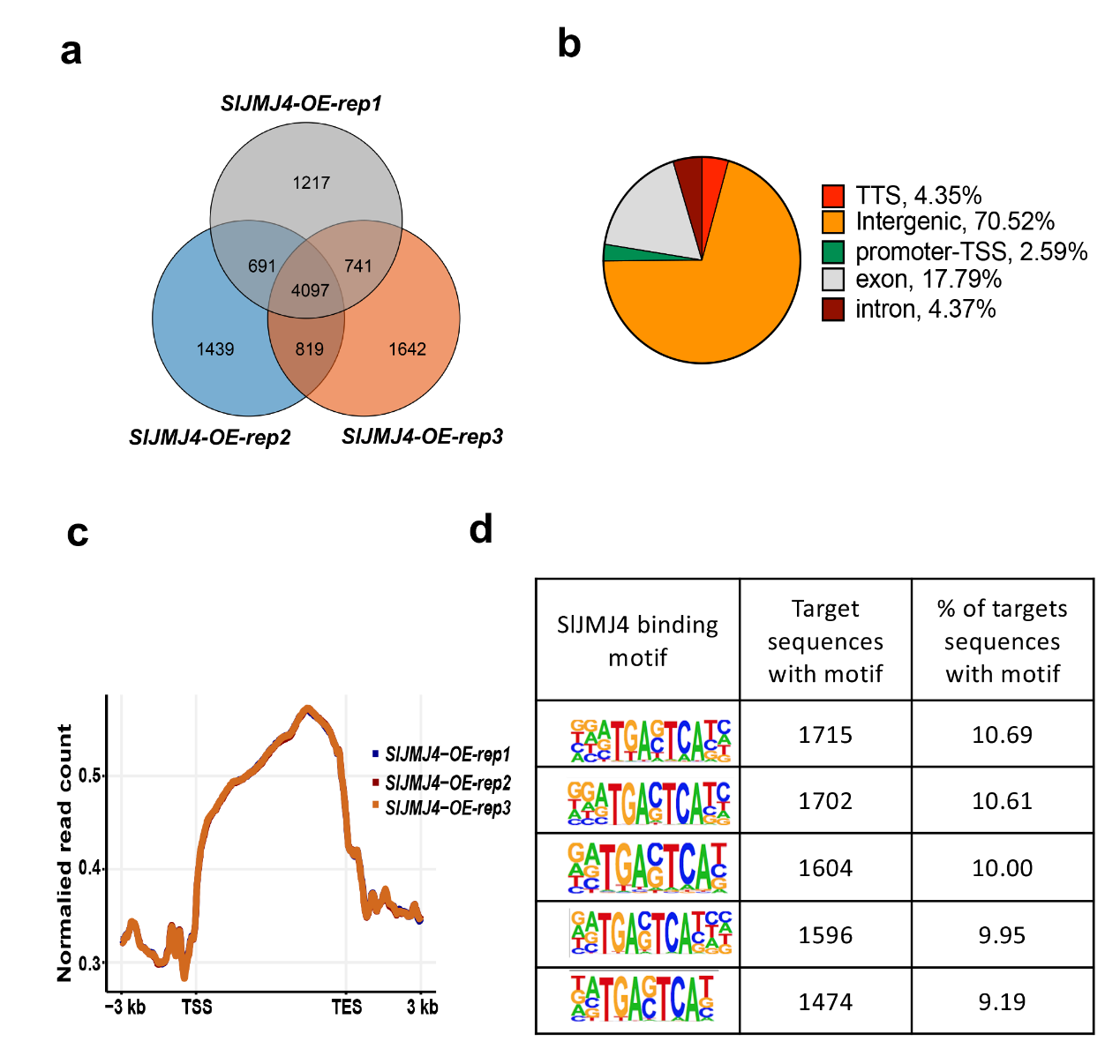


**Fig. S2.** Distribution of the SlJMJ4 binding sites in tomato genome. (**a**) Chromatin immunoprecipitation followed by sequencing (ChIP-seq) reveals 4097 shared genes in three biological replicates. (**b**) Distribution of SlJMJ4 binding sites in different regions of annotated genes. (**c**) Read density analysis further showed that these SlJMJ4 binding sites were uniformly distributed between the transcription start site (TSS) and the transcription end site (TES). (**d**) Motif enrichment analysis demonstrated that SlJMJ4 bound to motifs of its potential targets.


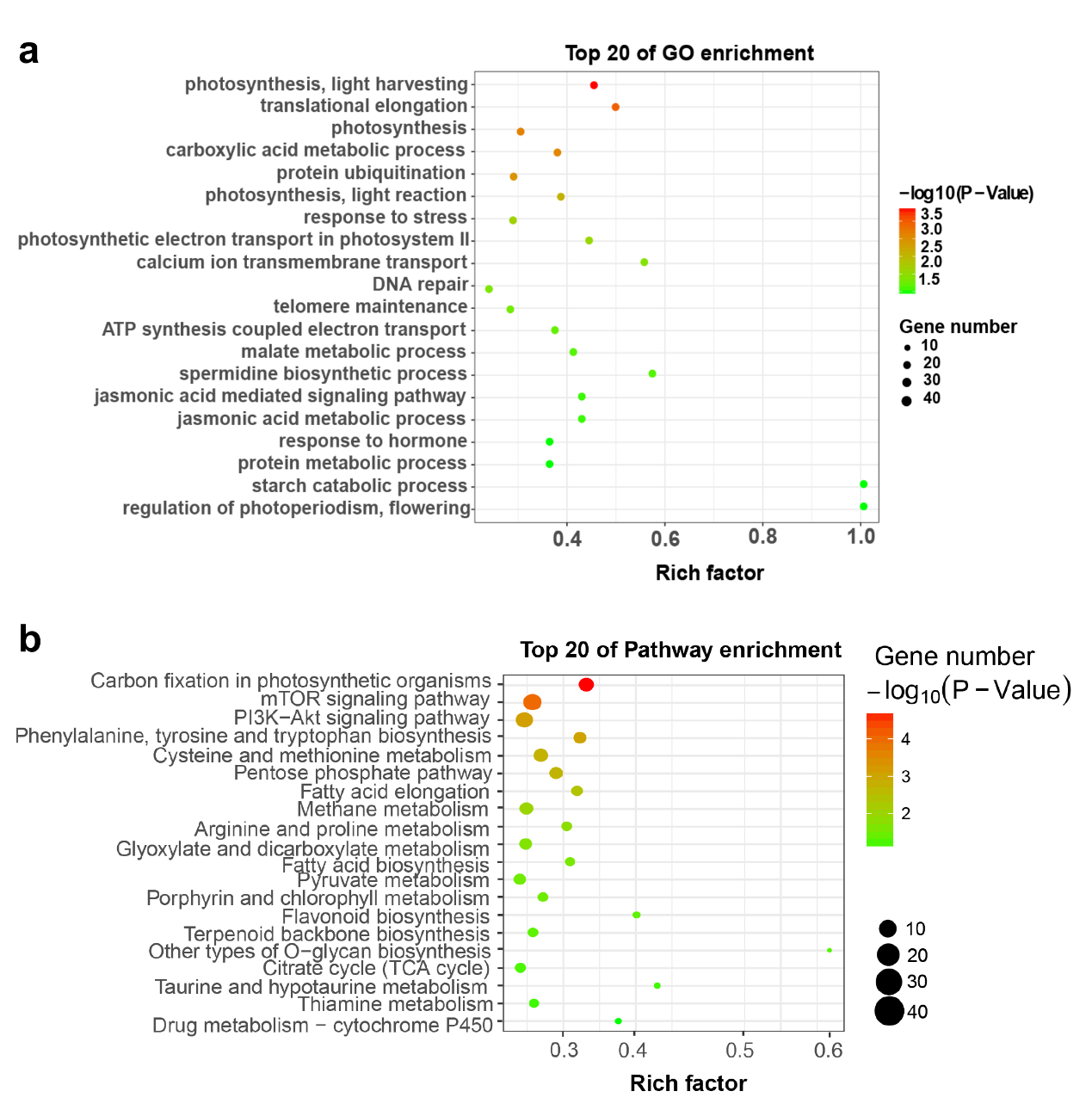


**Fig. S3.** GO (**a**) and KEGG pathway (**b**) functional clustering analysis of the gene clusters with binding of SlJMJ4.


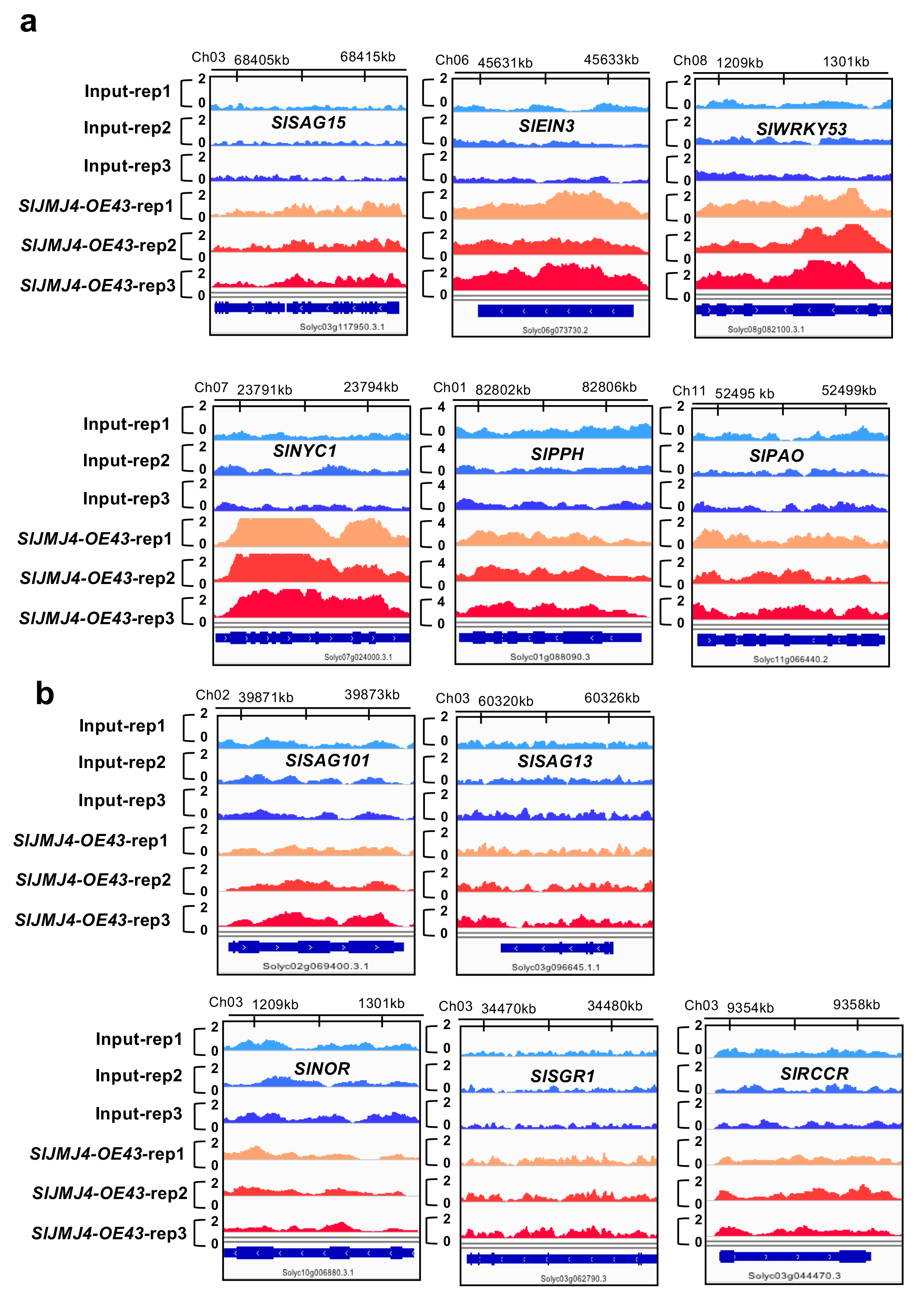


**Fig. S4.** Genome browser visualization of the binding sites of *SlSGA15, SlEIN3, SlWRKY53, SlNYC1, SlPPH , SlPAO, SlSAG101, SlSAG13, SlNOR, SlSGR and SlRCCR* genes detected by ChIP-seq in the *SlJMJ4-OE* line.
